# High concentration of blood lead levels among young children in Bagega community, Zamfara – Nigeria and the potential risk factor

**DOI:** 10.11694/pamj.supp.2014.18.1.4264

**Published:** 2014-07-21

**Authors:** Olufemi Olamide Ajumobi, Ahmed Tsofo, Matthias Yango, Mabel Kamweli Aworh, Ifeoma Nkiruka Anagbogu, Abdulazeez Mohammed, Nasir Umar-Tsafe, Suleiman Mohammed, Muhammad Abdullahi, Lora Davis, Suleiman Idris, Gabriele Poggensee, Patrick Nguku, Sheba Gitta, Peter Nsubuga

**Affiliations:** 1 Nigeria Field Epidemiology and Laboratory Training Programme, Abuja, Nigeria; 2 Ministry of Health, Gusau, Zamfara state, Nigeria; 3 Ministry of Environment, Gusau, Zamfara state, Nigeria; 4 Department of Community Medicine, Ahmadu Bello University, Zaria, Nigeria; 5 African Field Epidemiology Network, Kampala, Uganda; 6 Global Public Health Solutions, Atlanta, USA

**Keywords:** Lead poisoning, outbreak, child mortality, Nigeria

## Abstract

**Introduction:**

In May 2010, lead poisoning (LP) was confirmed among children <5years (U5) in two communities in Zamfara state, northwest Nigeria. Following reports of increased childhood deaths in Bagega, another community in Zamfara, we conducted a survey to investigate the outbreak and recommend appropriate control measures.

**Methods:**

We conducted a cross-sectional survey in Bagega community from 23rd August to 6th September, 2010. We administered structured questionnaires to parents of U5 to collect information on household participation in ore processing activities. We collected and analysed venous blood samples from 185 U5 with LeadCare II machine. Soil samples were analysed with X-ray fluorescence spectrometer for lead contamination. We defined blood lead levels (BLL) of >10ug/dL as elevated BLL, and BLL ≥45ug/dL as the criterion for chelation therapy. We defined soil lead levels (SLL) of ≥400 parts per million (ppm) as elevated SLL.

**Results:**

The median age of U5 was 36 months (Inter-quartile range: 17-48 months). The median BLL was 71µg/dL (range: 8-332µg/dL). Of the 185 U5, 184 (99.5%) had elevated BLL, 169 (91.4%) met criterion for CT. The median SLL in tested households (n = 37) of U5 was 1,237ppm (range: 53-45,270ppm). Households breaking ore rocks within the compound were associated with convulsion related-children's death (OR: 5.80, 95% CI: 1.08 - 27.85).

**Conclusion:**

There was an LP outbreak in U5 in Bagega community possibly due to heavy contamination of the environment as a result of increased ore processing activities. Community-driven remediation activities are ongoing. We recommended support for sustained environmental remediation, health education, intensified surveillance, and case management.

## Introduction

The World Health Organisation (WHO) estimates that 0.6% global burden of diseases and 600,000 causes of intellectual disability in children are due to lead poisoning. Common sources of lead are contaminated air, water, soil, food, consumer products, and mining activities. Lead is toxic to many organs of the body, particularly in children, causing permanent learning, behavior disorders, slowed growth, convulsions, and death [1–5]. A significant number of cases of lead poisoning are attributed to hand-to-mouth behavior, inhalation of combustion of leaded gasoline [6–8]. According to the guidelines of the United States (US) Centers for Disease Control and Prevention (CDC), an elevated blood lead level (BLL) is defined as ≥10 µg/dL [9]. The World Health Organization (WHO) has adopted a critical level of 10-15 µg/dL [10]. The major treatments for lead poisoning are removal of source of lead and chelation therapy.

Reports of massive deaths of sick children with non-specific symptoms in two villages Dareta and Yargalma in Anka and Bukkuyyum local government areas (LGAs) of Zamfara state, northwest Nigeria have been linked to outbreak of acute lead poisoning due to illegal mining of gold-rich ore [11]. About 80% of the children had convulsions and died as a result of lead poisoning [11]. Until then, lead poisoning in children was rare and had never been linked to mining activity in Nigeria. Other villages with suspected cases of lead poisoning were placed under surveillance and an educational campaign which discourages ore processing activities within the community but encourages the need for testing and environmental remediation were implemented.

On 21st August 2010 Zamfara State rapid response team reported recent unexplainable deaths in children below 5 years (U5) from Bagega community and hypothesised that this was probably due to suspected lead poisoning. Reports of active gold mining activity within this community were unverified. Bagega was not one of the communities under surveillance for possible lead contamination and until then no incidents of acute lead poisoning had been confirmed in this community. We investigated a suspected outbreak of lead poisoning in Bagega community Zamfara State, to determine the proportion of U5 with lead poisoning, to identify children needing chelation therapy, determine risk factors, and assess the environment for lead contamination.

## Methods

### Study area

We conducted a cross sectional study in Bagega community, Zamfara State-Nigeria from 23 August to 6 September, 2010. Bagega community comprises eight settlements: Magaji, Galadima, Mayana, Sabon Fegi, Kurar Mota, Sanlawa, Rawaiyya and Guraguri with an estimated population of 2,450 people and 380 U5. Majority of the inhabitants were farmers while a significant proportion of youths earned a living by engaging in illegal mining of gold ore.

A suspected case of lead poisoning was defined as any U5 residing in Bagega, with vomiting, abdominal pain, headache or convulsion from March 2010 who failed to respond to antimalarial treatment or antibiotics [11]. A confirmed case was any resident with a laboratory confirmation of lead in blood above 10ug/dL assessed by LeadCare II machine. A census of all children (n = 400) was conducted and all eligible children aged 6-59 months (n = 185) whose caregivers gave informed consent were included.

Paediatric hospital surveillance records were reviewed for a period of 6 months prior to the survey. We interviewed and observed health staff at Bagega health centres and miners while performing their duties. We conducted a door-to-door survey and administered two part structured questionnaires to caregivers of U5 with sections for caregivers and mining operations/environmental exposures. We collected information on demographic characteristics, clinical history, potential sources of lead exposure in the home environment and household participation in ore processing activities. Global positioning system (GPS) coordinates were taken at the entrance of each household. The environment was observed for mining activity and presence of grinding machines for ore rocks.

### Blood samples: laboratory investigation

All U5 were selected. Venous samples were taken from 185 children U5 residing in 158 households whose parents gave consent. Blood samples were analysed with portable LeadCare II machine (ESA Biosciences, Chelmsford, MA 01824, USA) at the Blood Lead/Inorganic Metals Laboratory, General Hospital Gusau, Zamfara State. This machine has an analytic range of <3.3-65 µg/dL. The laboratory environment was properly kept from dust and at room temperature, and powder free gloves were worn to prevent interference with analysis of blood samples by the LeadCare II machine. The machine was used according to the manufacturer's instructions. The LeadCare II analyser was calibrated appropriately for the LeadCare II blood lead test kits lot in use. Quality control tests were performed before and after each batch of the analysis. We defined blood lead levels (BLL) of >10ug/dL as elevated BLL, and BLL ≥45ug/dL as the criterion for chelation therapy. In cases where there were BLL > 65µg/dl (beyond the capability of the LeadCare II analyser), the blood sample was diluted serially using diluted 0.34M Hydrochloric acid.

### Environmental samples

Soil samples were taken from the community including ore processing areas and within randomly selected compounds in households, and analysed with hand-held X-ray fluorescence (XRF) spectrometer analyser (Innov-X systems, Inc., MA 01801, USA) for lead contamination. We defined soil lead levels (SLL) of ≥400 parts per million (ppm) as elevated SLL.

### Data analysis

Data collected were analysed with Epi Info version 3.5.3 software[12]. The outbreak was characterised in time, place, and person. Frequency tables were generated. Various forms of exposures to lead in live children without history of convulsion and in children whose deaths were preceded by history of convulsion were compared by chi square test. Odds ratio was calculated. Proportions were expressed at 95% confidence interval (CI).

### Ethical consideration

This was public health intervention in response to an outbreak. A non-research determination was sought and letter obtained from Federal Ministry of Health. Informed consent was obtained from participants. Confidentiality of the given information was assured and maintained.

## Results

### Surveillance: Review of Bagega Primary Health Care (PHC) records

There were 14 deaths of U5 (including 7 deaths of children in a household) out of 17 recorded suspected/probable cases of lead poisoning, between 1st July, and 22nd August 2010. Record review prior to this period did not reveal death suggestive of lead poisoning. Six-month crude mortality rate and 1-month crude mortality rates were 75 per 1000 and 31 per 1000 population of U5 respectively. We witnessed the convulsion of an infant and 3 year old at the PHC Bagega whose BLL were found to be elevated.

### Household survey

Forty-five out of 158 households (28.5%) were participating in ore processing in the community while 38 households (24.1%) did so within their household (Table 1). The processes for processing gold from leaded ore in Bagega were described as breaking ore rocks, grinding ore rocks, washing, sluicing, washing with mercury (Hg), drying, amalgamation with mercury (Hg) and finally, melting. Figure 2 shows the frequency of participation of households in ore extraction activities. A miner said, “I make N100, 000 ($625) from selling a bag of ore rocks. The price of gold has increased tremendously”.

**Figure 1:**
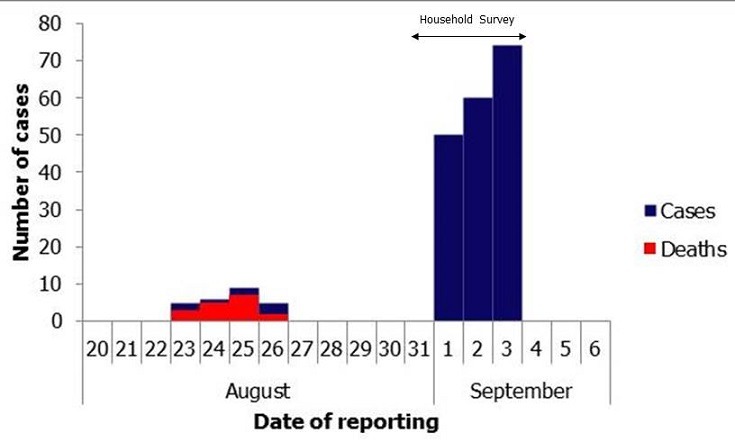
Distribution of Cases and Deaths in children during outbreak of Lead poisoning in Bagega, Zamfara State, Nigeria; September 2010

**Figure 2:**
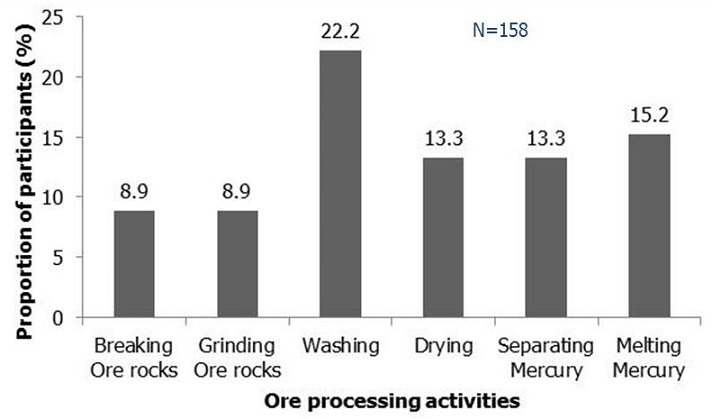
Participation of households in gold extraction activities in Bagega

**Table 1:** Lead levels in children and in soil, Bagega, Zamfara State, September 2010

Participation in ore processing (n = 158)	Number of households (%)
Outside the household	45 (28.5)
Within the household	38 (24.1)
Households (n = 37)	Number of Households with elevated SLL (%)
Elevated SLL (≥400ppm)	22 (59.5)
BLL (n = 184)	Number of U5 living in households with elevated SLL (%)
≥10µg/dL	63 (34.2)
≥45µg/dL	57 (31.0)
Ore processing sites (n = 6)	SLL (ppm)
Median	32, 240
Range	4180 - 100, 000

Of the 27 (6.3%) recorded deaths, 10 occurred in the 6 months preceding the survey. In all, 184 out of 185 U5 were confirmed to have lead poisoning. Figure 1 shows distribution of cases and deaths in children during outbreak of lead poisoning in Bagega. The median age of U5 was 36 months (inter-quartile range: 17-48 months). There were 87 males representing 47%. Fifty-three of the children (28.6%) had reportedly convulsed before and 35 (66%) of these had convulsions in the last 6 months. Households participating in breaking ore rocks within the compound were about six times more likely to have convulsion related-children's death (Table 2). Children who had convulsion in the previous 6 months were more likely to have died (p < 0.05).

**Table 2:** Association between ore processing activities, and convulsion related-deaths among U5, Bagega, Nigeria; September 2010

Exposure factors	Convulsed & died n = 19 (%)	Alive & nil convulsion n = 139 (%)	Odds Ratio (95%CI)
Breaking ore rocks	4 (21.1)	6 (4.3)	5.80 (1.08 - 27.85)
Grinding	2 (10.5)	6 (4.3)	2.59 (0.24 -16.05)
Washing	4 (21.1)	21 (15.1)	1.49 (0.33 - 5.34)
Drying	5 (26.3)	14 (10.1)	3.16 (0.77 -11.23
Separating mercury	3 (15.8)	16 (11.5)	1.44 (0.24 - 5.89)
Melting mercury	5 (26.3)	15 (10.8)	2.93 (0.72 - 10.28)

### Laboratory investigation

**Figure 3:**
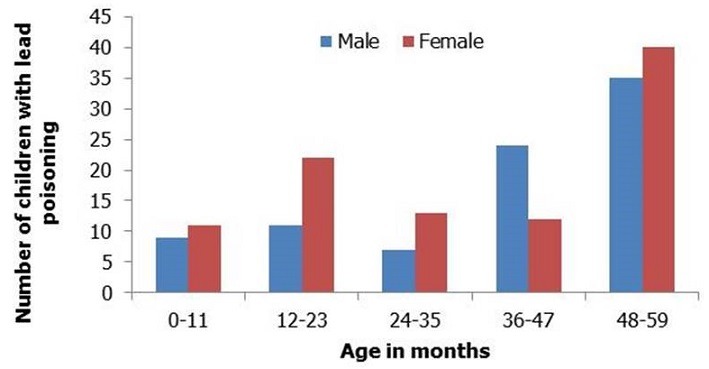
Age and gender distribution of U5 with lead poisoning, Bagega, Zamfara State, Nigeria; September, 2010

Blood samples from 185 (46.3%) U5 were examined. Of the 185 U5, 184 (99.5%) had elevated BLL >10ug/dL and 169 (91.4%) of them had severe lead poisoning of ≥ 45 µg/dL, which is the CDC-recommended level for initiating chelation therapy (Table 2). The median blood lead level was 71µg/dL with a range of 8-332µg/dL (Table 1). There were more females with lead poisoning compared to males across all the age groups except for ages 36 - 47 months (Figure 3). Sixty-three of the children (34.2%) with BLL ≥ 10µg/dL and 57 (31.0%) with BLL ≥ 45µg/dL resided in households with elevated soil lead levels (Table 1).

### Environmental assessment

Active and large-scale mining was ongoing in Bagega community during the time of the study. The activities undertaken ranged from breaking and grinding the ore, washing, sluicing (washing on a mesh under running water), to drying the ore and amalgamation with mercury and melting to produce gold. Processing area/mining market was about 4 hectares and had borders with large reservoir used by locals. Children were seen participating actively in all the stages of ore processing. There were more than 60 electric grinding machines (designed originally for grains) and >200 sluicing ponds. Miners were seen actively participating in processing ore rocks. The earth dam that serves the Bagega community (sometimes for domestic purposes and animal needs) was also used in the process of mining activity. In some instances, mining activity was being undertaken inside the dam. Animals were found grazing within the mining site. Two households were observed to have their ore dried in front of the compounds. A total of 37 soil sites were tested, with 14/37(37.8%) having lead level between 1000-10,000ppm, and 8/37 (21.6%) > 10,000ppm. The median soil lead level in the 37 tested households was 1,237ppm (range: 53-45,270ppm). Twenty-two households (59.5%) had elevated SLL, which exceeded the US Environmental Protection Agency standard of 400ppm, where children are present. Severe soil lead contamination at ore processing sites, with a median SLL of 32, 240 ppm and a range of 4,180 - 100,000 was found (Table 1).

## Discussion

We found that almost all the children younger than 5 years in Bagega who were screened in response to a suspected outbreak of lead poisoning in August/September 2010 had lead poisoning with the majority of them requiring chelation therapy. Our investigation further indicated that exposure to mined lead-containing ore dust results was responsible for high blood lead levels in the affected children.

Illegal gold mining was responsible for environmental contamination with lead, which led to increased childhood deaths observed in Bagega. This is corroborated by a previous study [11, 13]. This indicates a huge public health problem as hitherto, gold mining has not been commonly linked with lead toxicity unlike the case with mining of lead [14]. Environmental decontamination of affected compounds and enforcing laws prohibiting illegal gold mining will go a long way in addressing this problem.

This was a unique finding in a hitherto unexplored area, with magnitude of environmental contamination seven times those of areas investigated previously and remediated in Zamfara (personal communication, Nasir Tsafe Umar-Tsafe) and amongst few studies on lead poisoning linked with gold mining activities. Gold mining as cause of lead poisoning was an unexpected finding. Of importance is the relationship between lead poisoning and illicit community-based gold mining activities.

Children are exposed to lead dusts generated in ore processing activities. Though miners who participated in breaking ore rocks had increased exposure via inhalation but could have contributed more danger to children living in households because their clothes must have been laden with highly concentrated lead residual dusts and would further expose their kids while bonding on arrival from work and using such clothes for household chores. These probably contributed to increased risk of convulsion-related deaths in children [13]. Age of child, maternal participation in ore processing, primary source of water and SLL of the family compound have been found to be associated with child mortality [11].

Because of the perceived increase in the price of gold in the international markets, the youths in this community seized this opportunity to earn sufficient income while engaging in illicit gold mining activities[15]. The use of grain grinding machine also showed the extent of poverty in the community.

**Limitations:** Only children 185 U5, who represented 46.3% of a total of 400 children enumerated, were tested because of limited testing kits. It would have been more representative if all children and adults were tested in Bagega community and if contiguous communities such as Abare, Sunke and Tugur Daji were surveyed. Land areas outside the community were not tested for possible differential lead contamination.

## Conclusion

Lead contamination of soil was introduced into community through processing of gold ore with high lead content, which placed young children at high risk for lead exposure and development of lead poisoning. **Recommendations:** These are part of the larger ongoing interagency effort which includes environmental remediation, administration of chelation therapy to affected children and educational campaign against ore processing activities within the community. Environmental decontamination of affected compounds and enforcing laws prohibiting illegal gold mining will go a long way in addressing this problem. We recommended to Zamfara State Ministry of Health to provide support for intensified health education on lead poisoning, blood lead surveillance and training of health workers on case management for lead poisoning; and the State Ministry of Mines and Solid Mineral to promote safe mining practices and to Zamfara State Ministry of Environment to provide adequate support for environmental remediation and intensified soil lead surveillance. Federal government of Nigeria should enforce adherence to safe mining laws. A longitudinal study of chronic effects of lead poisoning in the affected children should be considered. The role of environmental surveillance in Field Epidemiology and Laboratory Training Programme strengthens the one health concept and reinforces interdisciplinary collaboration.
